# Rapid microwave-assisted synthesis of nitrogen-doped carbon quantum dots as fluorescent nanosensors for the spectrofluorimetric determination of palbociclib: application for cellular imaging and selective probing in living cancer cells

**DOI:** 10.1039/d2ra05759j

**Published:** 2023-01-31

**Authors:** Galal Magdy, Fathalla Belal, Heba Elmansi

**Affiliations:** a Pharmaceutical Analytical Chemistry Department, Faculty of Pharmacy, Kafrelsheikh University P.O. Box 33511 Kafrelsheikh Egypt galal_magdy@pharm.kfs.edu.eg +201000137394; b Pharmaceutical Analytical Chemistry Department, Faculty of Pharmacy, Mansoura University P.O. Box 35516 Mansoura Egypt

## Abstract

The current study introduces a spectrofluorimetric methodology for the assessment of palbociclib without the need for any pre-derivatization steps for the first time. This approach relied on the palbociclib quenching effect on the native fluorescence of newly synthesized nitrogen-doped carbon quantum dots (N-CQDs). An innovative, facile, and rapid microwave-assisted pyrolysis procedure was applied for the synthesis of N-CQDs using available and economic starting materials (the carbon source is orange juice and the nitrogen source is urea) in less than 10 minutes. Full characterization of the prepared QDs was carried out using various techniques. The prepared N-CQDs exhibited good fluorescence emission at 417 nm after excitation at 325 nm with stable fluorescence intensity and good quantum yield (29.3%). They showed spherical shapes and narrow size distribution with a particle size of around 2–5 nm. Different experimental variables influencing fluorescence quenching were examined and optimized. A good linear correlation was exhibited alongside the range of 1.0 to 20.0 μg mL^−1^ with a correlation coefficient of 0.9997 and a detection limit of 0.021 μg mL^−1^. The proposed methodology showed good selectivity allowing its efficient application in tablets with high percentage recoveries and low percentage RSD values. The mechanism of quenching was proved to be static by applying the Stern–Volmer equation at four different temperatures. The method was validated in accordance with ICHQ2 (R1) recommendations. Intriguingly, N-CQDs demonstrated good biocompatibility and low cytotoxicity, which permitted cellular imaging and palbociclib detection in living cancer cells. Therefore, the proposed method may have potential applications in cancer therapy and related mechanism research.

## Abstract 2

The current study introduces a spectrofluorimetric method for the estimation of palbociclib depending on a novel fluorescent carbon quantum dots-based nanosensor without the need for any pre-derivatization steps for the first time.

## Graphical abstract

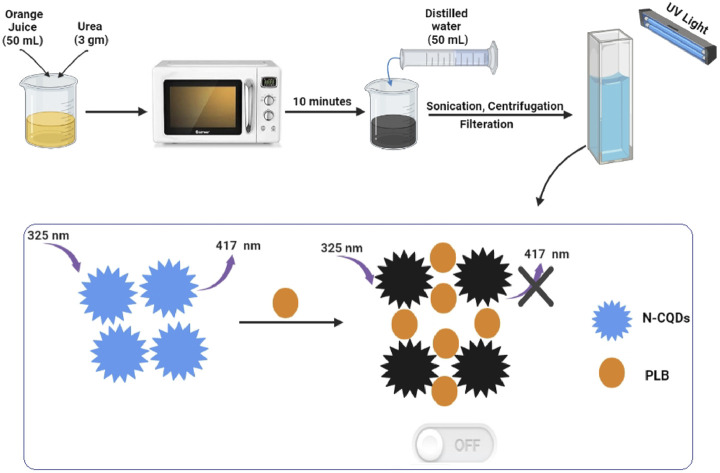

## 1. Introduction

**Fig. 1:**
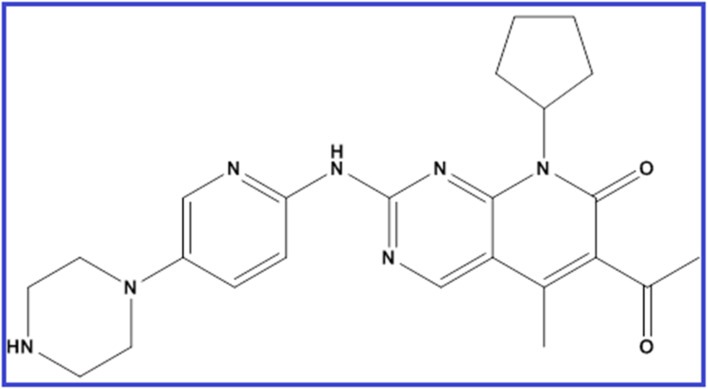
Chemical formula of PLB.

Palbociclib (PLB) is 6-acetyl-8-cyclopentyl-5-methyl-2-[(5-piperazin-1-ylpyridin-2-yl)amino]pyrido[2,3-d]pyrimidin-7-one (Fig. 1).^1^ It is a pyridopyrimidine, a secondary and tertiary amino compound, and a member of cyclopentanes. As breast cancer is considered the second most common cancer globally and the most common cancer in women,^2^ the synthesis and analysis of new anti-breast cancer drugs is of urgent need. PLB has been approved recently by FDA for the management of endocrine-resistant metastatic breast cancer combined with endocrine therapy.^3^ In 2015, the randomized phase II PALOMA-1 trial defined for the first time the activity and efficacy of PLB as an anti-breast cancer medication inhibiting cyclin-dependent kinases.^3^ This targeted therapy is promising and effective for stopping cancer progression.^4^ It is sometimes combined with other drugs as an aromatase inhibitor or fulvestrant. PLB is available as Ibrance^®^ capsules or film-coated tablets in different doses of 75, 100, and 125 mg.

Few methods have been reported for the determination of PLB in pharmaceutical dosage forms and biological samples, mainly using UPLC,^5^ HPLC,^6,7^ and LC-MS/MS^8,9^ methods. As far as we know, no spectrofluorimetric methods have yet been adopted for its determination.

The construction of a novel multifunctional fluorescence platform as carbon quantum dots (CQDs) is gaining great attention in the determination of different pharmaceutical compounds, metals, and biological compounds, as well as cellular imaging.^10–21^ They are similar in size and photo-electrochemical properties; however, they vary in the internal structure and surface chemical groups. They are mono-disperse spherical nanoparticles with a carbon-based skeleton and a large number of oxygen-containing groups on the surface.^22^ Additionally, heteroatom-doped CQDs were developed for enhancing the electrical and optical characteristics of CQDs. Fluorine, boron, sulfur, phosphorus, and nitrogen are the commonly presented doping chemical elements.^21,23,24^ Different approaches have been reported for the synthesis of CQDs, including hydrothermal synthesis,^20,24–26^ microwave-assisted synthesis,^27–29^ chemical oxidation,^30^ and carbonizing organics methods.^31^

To date, no spectrofluorimetric methods have been reported for PLB assay and the published methods for its estimation require high-cost instruments and a large amount of organic solvents. Therefore, the main objective of this study was to construct a novel spectrofluorimetric method for the determination of PLB based on the merits of the quantum dots including, biocompatibility, good luminescence, facile synthesis, cost-effective starting materials, water-solubility, low toxicity levels, high sensitivity, and easy measurements.^32^ In the current work, nitrogen-doped carbon quantum dots (N-CQDs) were prepared by a rapid and facile microwave-assisted pyrolysis approach in less than 10 minutes utilizing orange juice (as a carbon source) and urea (as a nitrogen source) for the first time. Herein, PLB quantitatively quenches the fluorescence of the prepared quantum dots. This quenching was investigated in order to design a spectrofluorimetric method for its estimation. The novelty of this study is addressed as being the first spectrofluorimetric approach for the determination of PLB without the need for any pre-derivatization steps or sophisticated techniques. Since the studied drug does not exhibit native fluorescent properties, the importance of the proposed study is magnified. In addition to the outstanding features of N-CQDs, they demonstrated good biocompatibility and low cytotoxicity, which permitted cellular imaging and PLB detection in living cancer cells. Consequently, the developed method is expected to have substantial significance and potential applications in cancer therapy.

## 2. Experimental

### 2.1. Instruments

- A double-beam spectrophotometer (PG Instrument, UK) was utilized in spectrophotometric measurements.

- A Cary Eclipse fluorescence spectrophotometer operated with a Xenon flash lamp from Agilent Technologies (Santa Clara, CA 95051, United States) was used. It was operated at 750 V.

- All pH measurements were performed using a Jenway pH-meter 3510 (Jenway, UK).

- FT-IR spectra were obtained using the Thermo-Fisher Scientific Nicolet – iS10 FT-IR spectrometer (Thermo Fisher Scientific, Waltham, MA, USA). The instrument had a Ge/KBr beam splitter and a 4000 to 1000 cm^−1^ DTGS detector. The measurements were acquired with a resolution of 4 cm^−1^ in 32 scans.

- The morphology of N-CQDs was studied using a JEM-2100 high-resolution transmission electron microscope (HRTEM) (JEOL, Tokyo) operating at 200 kV.

- An ultrasonic bath (SS 101H 230, USA).

- A cooling centrifuge (2–16P, Germany).

- A vortex mixer (IVM-300p, Taiwan).

- Membrane filters (0.45 μm, Phenomenex, USA).

- A domestic Microwave (GE614ST, 2800 W, 2450 MHz, Samsung, Malaysia).

### 2.2. Materials, reagents, and chemicals

- Palbociclib was obtained from Pfizer, Freiburg, Germany.

- Urea, Britton-Robinson buffer, and methanol were purchased from Sigma Aldrich (St. Louis, MO, USA).

- HepG2 cell line was obtained from Nawah Scientific Company, Egypt.

- All materials and reagents were of analytical grade.

- Double distilled water was utilized throughout the work.

### 2.3. Preparation of stock solution and buffers

- A stock solution of PLB at a concentration = 100.0 μg mL^−1^ was prepared in methanol. Subsequent dilutions of the stock solution were prepared using double-distilled water. The solutions remained stable for at least 14 days then they were kept in the fridge.

- A Britton-Robinson buffer (0.02 M) was prepared in distilled water to cover pH ranges from 2–12.

### 2.4. Fabrication of N-CQDs

**Scheme 1:**
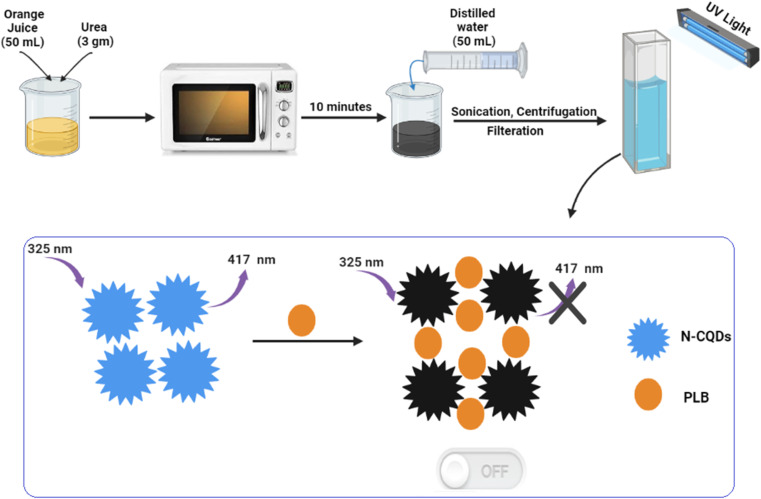
Synthesis of N-CQDs and application for determination of PLB.

The preparation of N-CQDs was carried out by dissolving 3 gm of urea in 50 mL of orange juice and then heating it for 10 minutes in a domestic microwave till it was completely charred. Next, the product was left to cool, diluted with water to 100 mL, and centrifuged at 6000 rpm for 15 min to eliminate suspended particles. The clear layer was filtered and the volume was adjusted to 200 mL with water to prepare the QD stock solution. The working solution was obtained by transferring 10 mL of QD stock solution into a 100 mL volumetric flask and completing to mark with double-distilled water (Scheme 1). The prepared solutions were kept for further use in the refrigerator.

### 2.5. Spectrofluorimetric measurements

After optimizing different parameters, in a set of 5 mL measuring flasks, 2 mL Britton–Robinson buffer (pH 2, 0.02 M) was added, followed by the addition of 125 μL of N-CQDs. Then, aliquots of the studied drug over the range (1.0 to 20.0 μg mL^−1^) were added and the flasks were completed with distilled water up to the mark. The fluorescence measurements were performed at room temperature at 325/417 nm as the excitation/emission wavelengths, respectively. Quenching of the fluorescence was performed and plotted *vs.* the drug concentration in μg mL^−1^ to construct the calibration curve and carry out the regression analysis.

### 2.6. Quantum yield measurements

The quantum yield (QY) of N-CQDs was determined by adopting the single point method using eqn 1:^33,34^

1 *Φ*_N-CQDs_ = *Φ*_QS_ × (*F*_N-CQDs_/*F*_QS_) × (*η*_N-CQDs_/*η*_QS_)^2^ × (*A*_QS_/*A*_N-CQDs_)

where: *F* is the integrated measured emission intensity, *Φ* denotes QY, *A* represents the absorbance, and *η* represents the refractive index of the solvent.

Quinine sulfate (QS) was prepared in 0.1 M H_2_SO_4_ and employed as the standard (QY: 0.54 at 350 nm). In the aqueous solutions *η*_N-CQDs_/*η*_st_ = 1.

### 2.7. Assay of pharmaceutical preparation

A set of laboratory-prepared PLB tablets (Ibrance^®^ Tablets, 100 mg PLB/Tablet) were obtained by mixing with 10 mg each of microcrystalline cellulose, lactose monohydrate, colloidal anhydrous silica, sodium starch glycolate type A, and magnesium stearate while maintaining the drug's pharmaceutical concentration (100 mg PLB/Tablet). An amount of the powder corresponding to 100.0 mg PLB was transferred into a small flask and 50 mL of methanol was added. The flask was subjected to sonication in an ultrasonic bath for 15 min. The solution was filtered in a clean dry 100 mL measuring flask and filled with the same solvent to the volume. Finally, volumes within the linear range (4.0, 8.0, 12.0, 16.0 μg mL^−1^) were moved into a 5 mL set of measuring flasks and completed with double-distilled water. The steps mentioned in Section 2.5 were followed and percentage recoveries were computed from the calibration plot or the derived regression equation.

### 2.8. Cell lines and reagents for MTT assay

HepG2 cells were grown in DMEM (Biowest, France) and supplemented with 100 IU mL^−1^ penicillin/streptomycin (100 μg mL^−1^) (Lonza, 17-602E) and with bovine serum albumin (10%, Life Science Group L, UK, Cat No: S-001B-BR). PLB (Quencher) or doxorubicin (control for the MTT experiment) was prepared in DMSO (10 mM stock).

### 2.9. Cell viability by MTT assay

The seeding of cancer cells was performed in a 96-well plate (100 μL per well). After overnight incubation at 37 °C and 5% CO_2_, the cells were incubated with either serial dilutions of the N-CQDs (0.03, 0.015, 0.0075, 0.00375, 0.001875, 0.0009375%) or doxorubicin (50, 25, 12.5, 6.25, 3.125, 1.65 μM). After incubation for 48 hours, 3-(4,5-dimethylthiazoyl)-2,5-diphenyl-tetrazolium bromide (MTT) (5 mg mL^−1^, phosphate-buffered saline (PBS)) was added, followed by incubation of the plate for 4 hours. Then, to dissolve the formazan crystals, an acidified sodium dodecyl sulphate (SDS) solution (10% SDS + 0.01N HCl in 1× PBS) was utilized. A Biotek plate reader (Gen5™) was used to measure the absorbance after 14 hours of incubation at λ_570-630_ nm.^35,36^

### 2.10. Cellular bioimaging

HepG2 cells were seeded on a coverslip in a 6-well plate (2 × 10^5^ cells/ml, 2 mL in each well). After overnight incubation, cells were treated with 0.01% of N-CQDs for 6 hours alone or with the quencher PLB (10 nM). After that, the cells were carefully washed with PBS and fixed with ice-cold methanol for 30 min at room temperature (RT). Untreated control cells were stained for 15 minutes at RT with 0.5 μM ethidium homodimer. After washing with PBS, the fixed cells were mounted on a glass slide. The coverslip was mounted on a glass slide and visualized using the Leica fluorescence microscope, Leica DMI 8, Leica Application Suite X (Leica, Germany).

## 3. Results and discussion

PLB is the first CDK4/6 inhibitor to be approved for use in humans. It has been widely used for the treatment of breast cancer. Therefore, our motivation was to investigate a novel method for its determination. As the heteroatom doping of CQDs has gained much attention, in this work, we demonstrate a simple, new, and rapid microwave-assisted pyrolysis strategy for N-CQDs synthesis using orange juice and urea as sources for carbon and nitrogen, respectively (Scheme 1). The prepared N-CQDs possess strong fluorescence emissions. Interestingly, this fluorescence could be selectively quenched by PLB, which could be a basis for an innovative methodology for its sensitive spectrofluorimetric analysis for the first time.

### 3.1. Characterization of N-CQDs

**Fig. 2:**
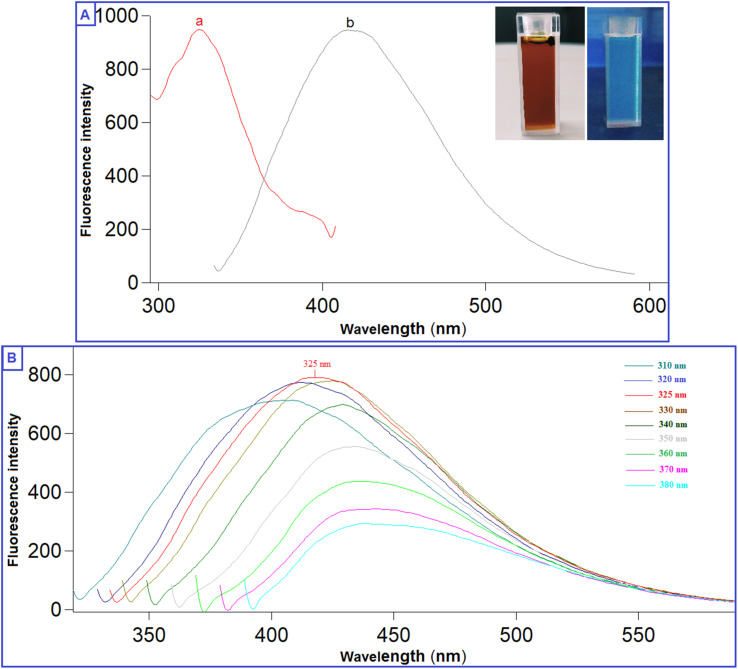
(A) Fluorescence spectra of N-CQDs; (a) excitation and (b) emission spectra (inset: photographs of N-CQDs under visible light and UV light), (B) Fluorescence spectra of N-CQDs at varied excitation wavelengths (310–380 nm).

**Fig. 3:**
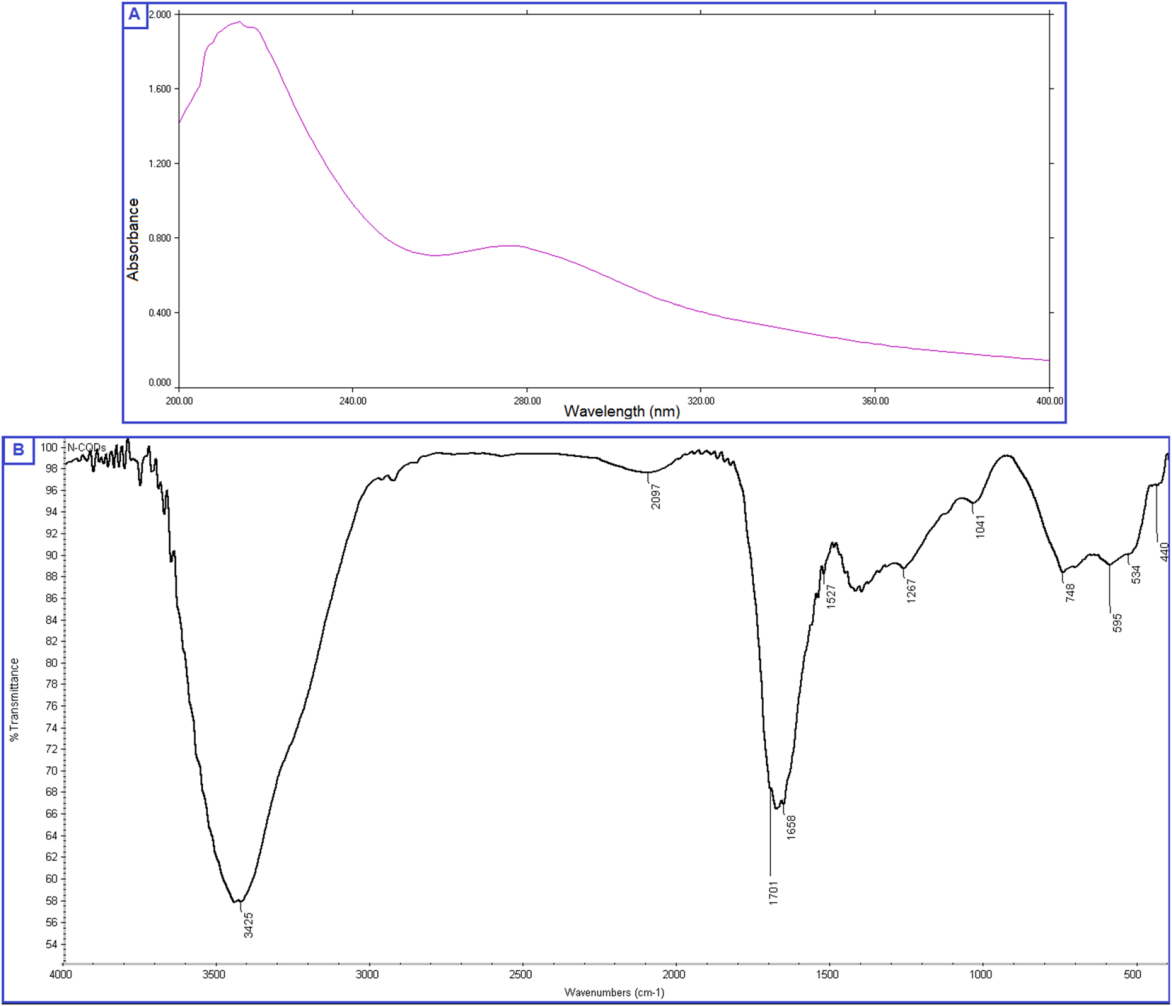
(A) UV absorption spectrum of N-CQDs, (B) FTIR spectrum presenting the surface functionality of N-CQDs.

**Fig. 4:**
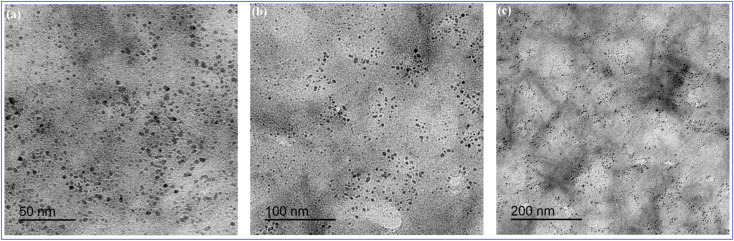
HRTEM images of N-CQDs; (a) 50 nm, (b) 100 nm, (c) 200 nm.

The prepared QDs were investigated by extensive characterization using different spectroscopic and microscopic techniques. The optical images of the N-CQDs solution under UV light and visible light are presented in Fig. 2A. The solution of N-CQDs demonstrated a dark orange color in visible light and a strong blue fluorescence under the UV light with a long-lasting homogenous phase, no obvious precipitation was seen, and stable fluorescence intensity for more than four weeks was observed. Spectrofluorimetric measurements showed that QDs exhibited high fluorescence intensity at 417 nm following excitation at 325 nm (Fig. 2A) and displayed a high QY (29.3%) utilizing QS as a reference. The emission of the synthesized QDs demonstrated excitation dependency across the 310 to 380 nm range and the optimum fluorescence emission was obtained at 325 nm, as presented in Fig. 2B. The UV spectrum (Fig. 3A) was scanned to inspect the optical features of QDs and two characteristic bands were recorded at *λ*_max_ of 213 and 275 nm, equivalent to π–π*/n–π* transitions.^37,38^ FTIR was also incorporated to examine the surface functional groups of N-CQDs. Finally, the obtained spectrum revealed the following peaks: O–H/N–H (3500–3100 cm^−1^), C–N (2097 cm^−1^), C=O (1701 cm^−1^), C=C (1658 cm^−1^), and C-H (595 cm^−1^) (Fig. 3B).^39^ In addition, HRTEM images indicated that N-CQDs were well separated without any apparent aggregation with spherical shapes and sizes in the range of 2–5 nm (Fig. 4).

### 3.2. Investigation of the quenching mechanism of N-CQDs

**Fig. 5:**
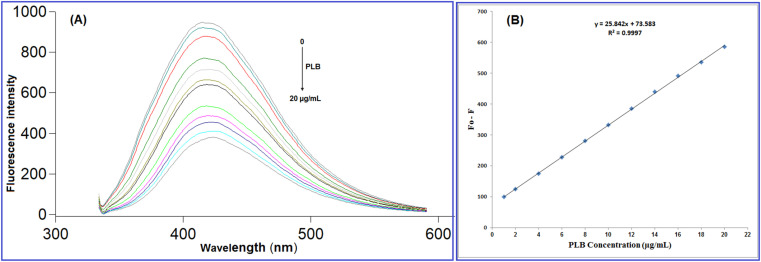
(A) Fluorescence emission spectra of N-CQDs after addition of different PLB concentrations (from top to bottom: 0, 1.0, 2.0, 4.0, 6.0, 8.0, 10.0, 12.0, 14.0, 16.0, 18.0, 20.0 μg mL^−1^), (B) The linearity plot of fluorescence quenching against PLB concentration.

As illustrated in Fig. 5, upon adding increased concentrations of PLB, the native fluorescence of N-CQDs was quantitatively quenched, which can be referred to as damage to the surface passivation layer of QDs by the cited drug.^40,41^ The fluorescence quenching mechanisms include two main categories: dynamic and static. The difference between dynamic and static quenching can be examined by lifetime observations or, better yet, their temperature dependency. In dynamic quenching, higher temperature settings cause faster diffusion and a rise in the Stern–Volmer quenching constant (*K*_SV_), while higher temperatures cause the complexes to dissociate and the quenching constant to decrease in static quenching.^42^ To determine the potential quenching mechanism, the Stern–Volmer equation (2) was employed:^43^

2 *F*_0_/*F* = 1 + *K*_sv_[*Q*] = 1 + *K*_q_*τ*_0_[*Q*]

Considering that F is the N-CQDs-PLB system fluorescence intensity and *F*_0_ is for N-CQDs only, *K*_SV_ is the Stern–Volmer quenching constant, [*Q*] is the PLB concentration, *k*_q_ denotes the quenching rate constant, and *τ*_0_ represents the average lifetime of the fluorophore (10^−8^ s).

**Fig. 6:**
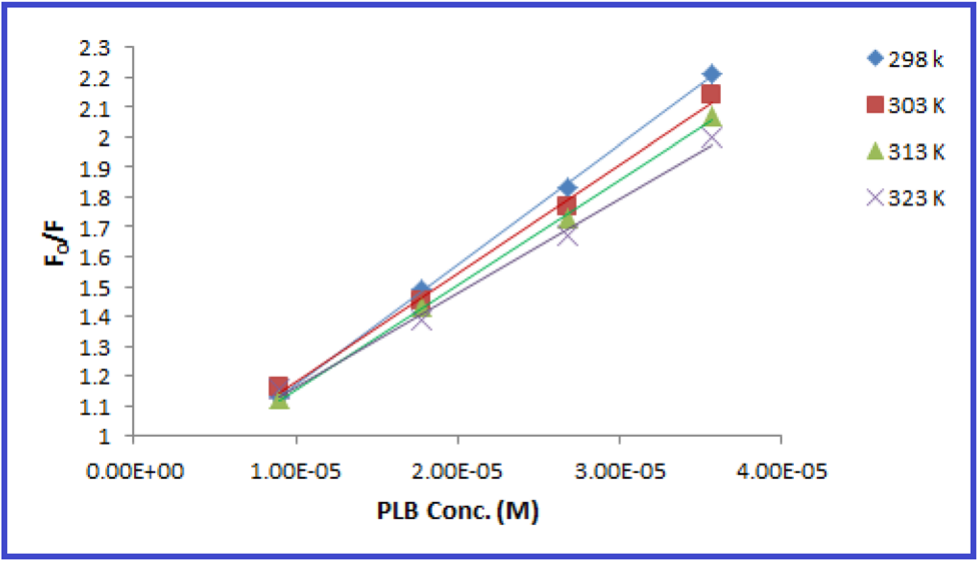
Stern–Volmer plots for the quenching of N-CQDs fluorescence by PLB at four different temperature settings (298, 303, 313, and 323 K).

**Fig. 7:**
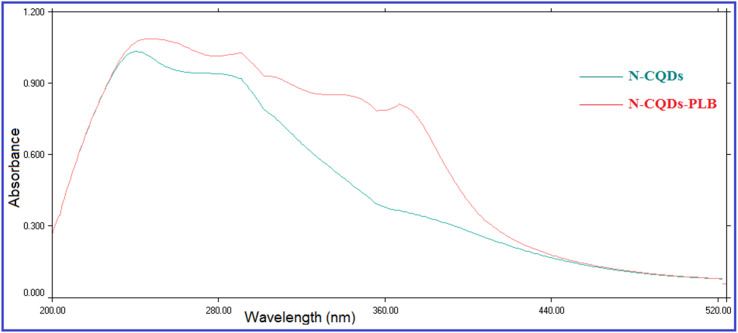
Effect of addition of PLB on the UV absorption spectrum of N-CQDs.

The fluorescence quenching efficiency (*F*_0_/*F*) was plotted against [*Q*] and *K*_SV_ values were calculated at four temperature settings (298, 303, 313, 323 K) (Fig. 6). *K*_sv_ values were found to be 4.01 × 10^4^, 3.641 × 10^4^, 3.506 × 10^4^, and 3.158 × 10^4^ L mol ^−1^ at 298, 303, 313, 323 K, respectively. As observed, *K*_sv_ values decreased upon increasing the temperature, indicating that the quenching proceeds by the static process. Additionally, from the obtained *K*_SV_ values, *k*_q_ values were calculated and were found to be 4.01 × 10^12^, 3.641 × 10^12^, 3.506 × 10^12^, and 3.158 × 10^12^ L mol^−1^ s^−1^, respectively, these values were significantly higher than the maximum diffusion rate constant (2.0 × 10^10^ L mol^−1^ s^−1^); further confirming the static quenching process.^44^ This mechanism includes the formation of N-CQDs/PLB non-emissive complex, as evidenced by changes observed in N-CQDs UV spectra after the addition of PLB. When PLB was added, a new absorption peak appeared at 370 nm, indicating the complex formation and confirming the static quenching mechanism (Fig. 7).

### 3.3. Optimization of experimental parameters influencing the fluorescence sensing

In order to reach the maximum sensitivity of the method, different parameters were studied including:

#### 3.3.1. Effect of pH

**Fig. 8:**
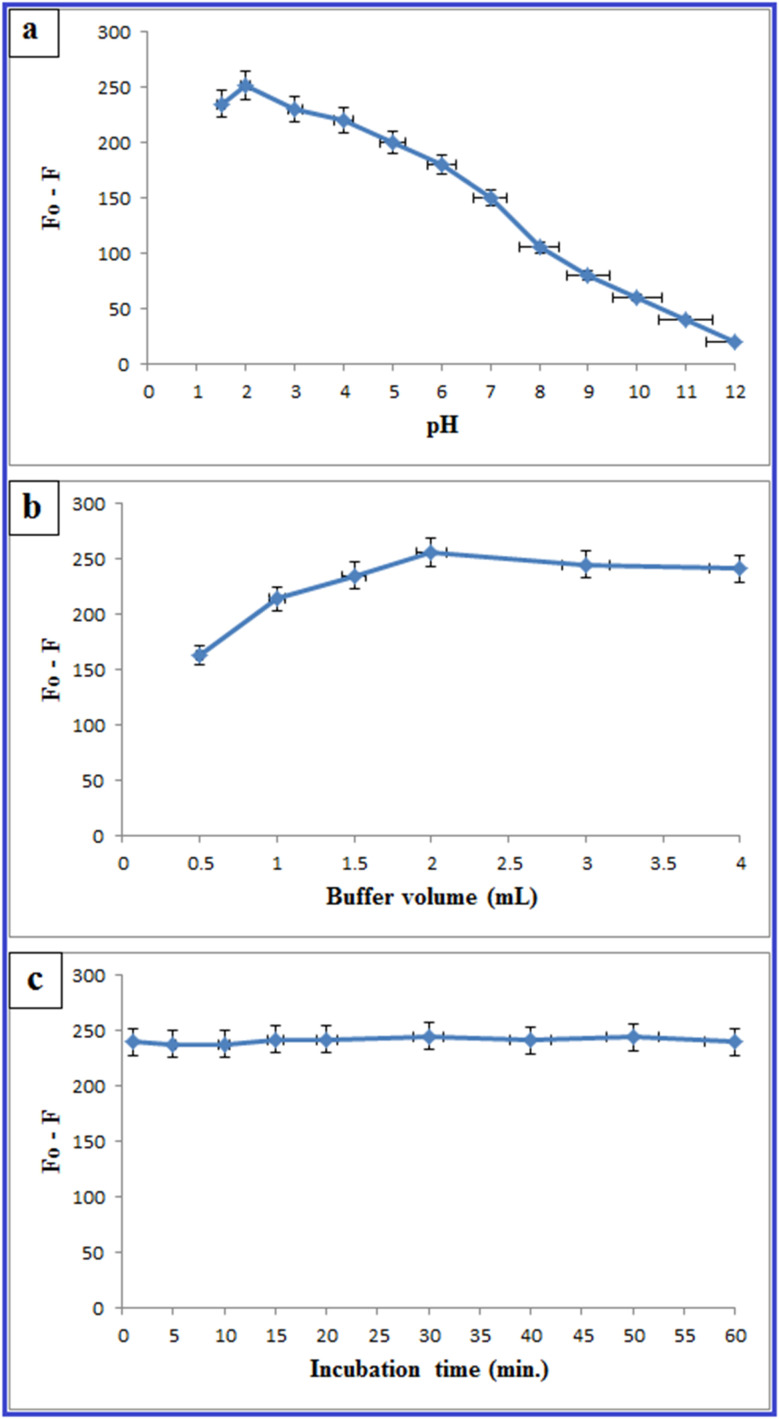
Effect of pH (a), buffer volume (b), and incubation time (c) on the quenching of the fluorescence spectrum of N-CQDs by PLB (6.0 μg mL^−1^).

The pH of a solution is well known to influence not just the fluorescence intensity of QDs, but also the interactions between QDs and target species.^45^ Using the Britton–Robinson buffer, the impact of the pH change on the quenching of N-CQD fluorescence by PLB was investigated. It was found that pH 2 was the ideal pH for fluorescence quenching (F_0_-F), and as pH increased, quenching decreased (Fig. 8a). As a result, the buffer volume was optimized utilizing several volumes in the range of (0.5–4.0 mL). The optimum volume for maximal fluorescence quenching with PLB was found to be 2 mL (Fig. 8b).

#### 3.3.2. Effect of incubation time

From 1 to 60 minutes, the incubation time influence on the interaction between N-CQDs and PLB was investigated. The reaction between PLB and N-CQDs was found to be fast, taking less than 1 min to complete, and the fluorescence signals were constant for more than 60 min, giving the suggested approach another advantage (Fig. 8c).

### 3.4. Validation of the method

The designed approach was validated in regard to the international council of harmonization guidelines (ICH).^46^ Different parameters were considered including; linearity, range, accuracy, precision, robustness, and selectivity.

The fluorescence quenching (*F*_0_ − *F*) was plotted against PLB concentration (μg mL^−1^) and a linear correlation was found in the range of 1.0 to 20.0 μg mL^−1^ (Fig. 5) with a linear regression equation can be represented as follows:

3 *F*_0_ − *F* = 25.84*C* + 73.58 (*r* = 0.9997)

where, *C* represents the concentration of PLB in μg mL^−1^. The analytical performance data for the developed methodology are shown in Table 1.

**Table 1:** Validation data for PLB by the proposed spectrofluorimetric method

Parameter	PLB
*λ*_ex_ − *λ*_em_	325–417 nm
Concentration range (μg mL^−1^)	1.0–20.0
Slope	25.84
Intercept	73.58
Correlation coefficient (*r*)	0.9997
S.D. of residuals (*S*_*y*/*x*_)	0.769
S.D. of intercept (*S*_a_)	0.168
S.D. of slope (*S*_b_)	0.037
Percentage relative standard deviation, % RSD	0.92
Percentage relative error, % error	0.27
Limit of detection, LOD a (μg mL^−1^)	0.021
Limit of quantitation, LOQ b (μg mL^−1^)	0.065

a LOD = 3.3*S*_a_/b.

b LOQ = 10*S*_a_/b, where *S*_a_ = standard deviation of the intercept and *b* = slope.

LOQ and LOD could be computed by referring to the mathematical equations: LOQ = 10*S*_a_/*b*, LOD = 3.3*S*_a_/*b*, where, *S*_a_ denotes the S.D. of the intercept of a regression line and *b* represents the slope. The results shown in Table 1 verify the acceptable sensitivity of the developed approach.

The method's accuracy was assessed using mean percentage recoveries and tested in triplicate runs with varied concentrations covering the PLB linearity range (Table 2). High recovery percentages (98.02–101.2%) were observed, demonstrating the method's high accuracy. Inter-day and intra-day precision were investigated at 3 concentration levels of PLB (4.0, 10.0, and 18.0 μg mL^−1^) and presented as % RSD and % error. The cited drug had low % RSD values (less than 1.12%) and % error (less than 0.65%), indicating that the developed approach was reasonably precise (Table 3).

**Table 2:** Application of the designed method for the determination of PLB in pure form

Parameter	Amount taken (μg mL^−1^)	Amount found (μg mL^−1^)	% Found^a^
	1.0	0.98	98.36
	2.0	1.99	99.49
	4.0	3.92	98.02
	6.0	5.98	99.59
	8.0	8.03	100.33
	10.0	9.99	100.00
	12.0	12.05	100.42
	14.0	14.14	101.00
	16.0	16.19	101.20
	18.0	17.89	99.41
	20.0	19.83	99.14
Mean			99.30
± S.D.			0.92
% RSD			0.922
% Error			0.27

a Average of 3 separate determinations.

**Table 3:** Precision data for the determination of PLB by the proposed method^a^

	Conc. (μg mL^−1^)	Intra-day^b^			Inter-day^c^		
		*x̄* ± S.D	% RSD	% Error	*x̄* ± S.D	% RSD	% Error
PLB	4.0	98.02 ± 0.86	0.865	0.49	99.31 ± 0.72	0.721	0.42
	10.0	100.21 ± 0.91	0.911	0.52	99.77 ± 1.04	1.038	0.59
	18.0	99.41 ± 1.12	1.121	0.65	100.34 ± 0.92	0.922	0.53

a Each reading is the average of three separate determinations.

b Within the day.

c Three consecutive days.

To investigate the robustness of the proposed approach, the impact of minor variations in the experimental factors concerning the fluorescence sensing of PLB was monitored. These factors include the volume of N-CQDs (125.0 μL ± 1), pH (2 ± 0.1), and buffer volume (2.0 mL ± 0.1 mL). It was verified that small changes did not significantly affect the quenching of the fluorescence intensities of N-CQDs by PLB, as presented in Table 4.

**Table 4:** Evaluation of robustness of the proposed method

Factor variation	PLB	
**1. Volume of N-CQDs**	**% Recovery** a	**% RSD**
**(125.0 μL ± 1)**		
124.0 μL	100.11	0.98
125.0 μL	99.54	0.93
126.0 μL	98.95	1.14

**2. pH**	**% Recovery** a	**% RSD**
**(2 ± 0.1)**		
1.9	99.52	0.91
2	99.62	0.85
2.1	100.23	0.79

**3. Buffer volume**	**% Recovery** a	**% RSD**
**(2.0 mL ± 0.1)**		
1.9 mL	99.81	1.08
2.0 mL	100.06	0.95
2.1 mL	99.75	1.19

a Each result is average of 3 separate determinations.

The suggested methodology was utilized to analyze PLB in its tablets with high percentage recoveries (98.07–101.73%) and low percent RSD values (1.561%) without interference from common excipients, demonstrating method selectivity (Table 5).

**Table 5:** Determination of PLB in prepared tablets by the proposed spectrofluorimetric method

Parameters	Amount taken (μg mL^−1^)	Amount found (μg mL^−1^)	% Found^a^
Prepared PLB tablets	4.0	3.98	99.63
	8.0	8.13	101.73
	12.0	11.76	98.07
	16.0	16.11	100.68
Mean			100.03
± S.D.			1.56
% RSD			1.561
% Error			0.78

a Each result is average of 3 separate determinations.

### 3.5. Method applications

#### 3.5.1. Analysis of PLB in tablets

PLB was determined using the designed approach in its tablet formulation. The concentrations of the cited drug were computed by referring to the regression equation. The % recoveries of the studied concentrations of PLB were acceptable (98.07–101.73%), as represented in Table 5, reflecting the method's selectivity in determining the cited drug with no interference from excipients.

#### 3.5.2. Cytotoxicity of N-CQDs and cellular imaging

**Fig. 9:**
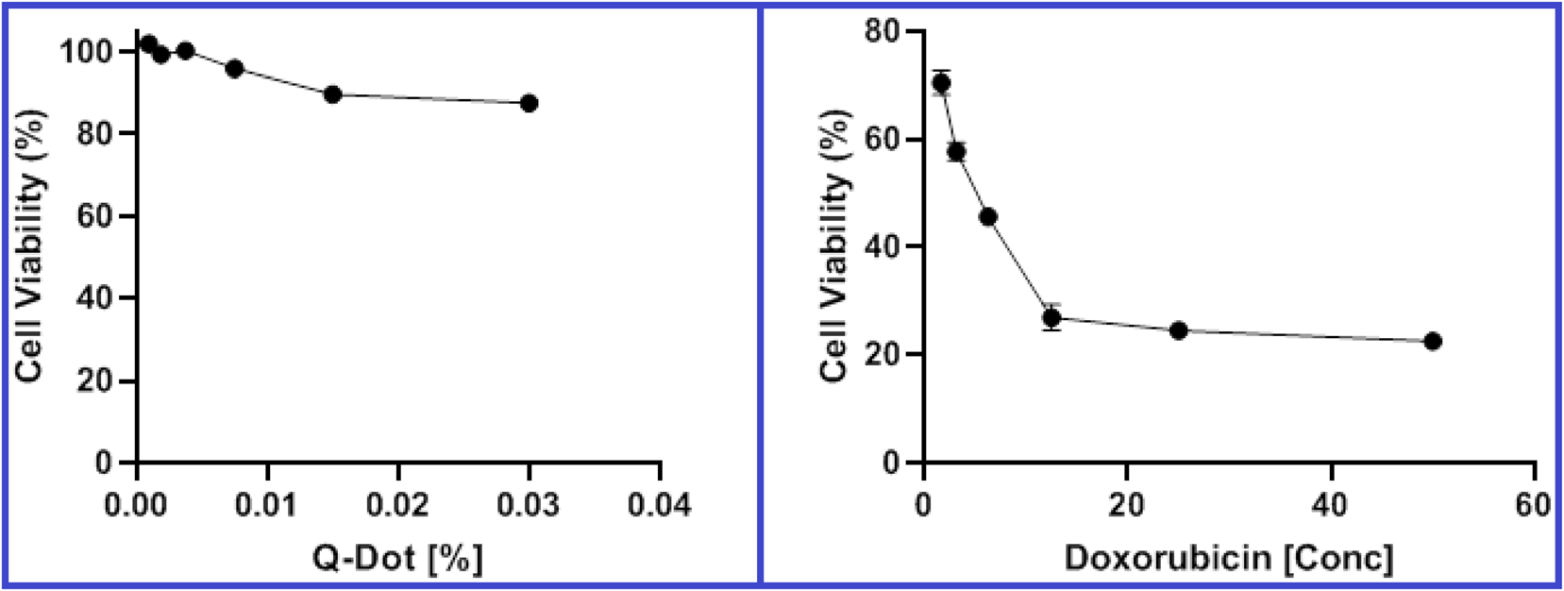
Viability of HepG2 cells after incubation for 48 hours with various concentrations of N-CQDs and doxorubicin as a control in the MTT assay.

**Fig. 10:**
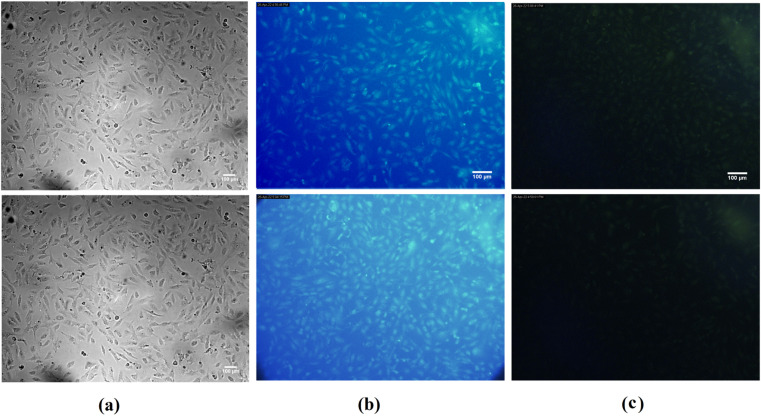
Confocal microscopy images of HepG2 cells incubated with 0.015% of N-CQDs for 6 hours. (a) Bright field images, (b) after excitation at 360 nm, and (c) after addition of 10 nM of PLB.

Before undertaking any biological applications, it is essential to assess the biocompatibility of N-CQDs. As shown in Fig. 9, the MTT assay outcomes indicated that N-CQDs showed good biocompatibility and low toxicity at the tested concentration range with IC_50_ of 0.2017% using doxorubicin as a control (Table 6). The viability of HepG2 cells was more than 85% even with a high concentration of N-CQDs after incubation for 48 hours. Therefore, the bioimaging experiment was performed. Fig. 10 shows the obtained confocal microscopy images of HepG2 cells where the cells exhibited no background autofluorescence by a laser with a 360 nm excitation. As can be observed, after incubating HepG2 cells with 0.015% of N-CQDs for 6 hours, the cells displayed blue fluorescence when excited at 360 nm. In addition, after incubation with the N-CQDs, no morphological damage to the cells was found, confirming their minimal cytotoxicity. All of these demonstrated that the N-CQDs were well suited to live-cell imaging. Based on the PLB-induced fluorescence quenching, N-CQDs were then used to detect PLB in living cells. Exogenous PLB was added to the N-CQDs-pretreated HepG2 cells. Fig. 10 indicates that no intracellular fluorescence was observed after adding 10 nM PLB to the growth medium for 6 hours at 37 °C. According to this result, the proposed N-CQDs could be employed to detect PLB in living cells as fluorescent probes. Consequently, the developed method could have substantial significance and prospective applications in cancer therapy.

**Table 6:** IC_50_ for the tested compounds

	N-CQDs	Doxorubicin
HepG2	0.2017%	5.08 μM

## 4. Conclusion

The current study introduces, for the first time, a sensitive and rapid spectrofluorimetric approach for the determination of PLB. The suggested approach relies on employing N-CQDs as fluorescent probes for the quantitation of the studied drug depending on the remarkable quenching effect of PLB on the fluorescence emission of N-CQDs without the need for any pre-derivatization steps. Orange juice as a carbon source and urea as a nitrogen source were used as available and economical starting materials for the rapid microwave-assisted synthesis of N-CQDs in less than 10 minutes. A good linear correlation was exhibited over the concentration range of 1.0 to 20.0 μg mL^−1^ with a detection limit of 0.021 μg mL^−1^ and a correlation coefficient of 0.9997. The designed system exhibited the merits of high selectivity and reproducibility as well as PLB assay in prepared tablets with satisfactory percentage recoveries (98.07–101.73%). Moreover, the N-CQDs demonstrated low cytotoxicity and high biocompatibility, permitting the cellular imaging and detection of PLB in living cells. ICHQ2 (R1) guidelines were used to validate the proposed technique.

## Author contributions

Galal Magdy: conceptualization, methodology, formal analysis, validation, data curation, visualization, writing–original draft. Fathalla Belal: conceptualization, resources, investigation, writing–review and editing. Heba Elmansi: conceptualization, data curation, investigation, writing–review and editing.

## Conflicts of interest

There are no conflicts of interest to declare.

## Supplementary Material
